# Trehalose-Bearing Carriers to Target Impaired Autophagy and Protein Aggregation Diseases

**DOI:** 10.1021/acs.jmedchem.3c01442

**Published:** 2023-11-30

**Authors:** Ali Maruf, Małgorzata Milewska, Máté Varga, Ilona Wandzik

**Affiliations:** † Department of Organic Chemistry, Bioorganic Chemistry and Biotechnology, Faculty of Chemistry, Silesian University of Technology, Krzywoustego 4, 44-100 Gliwice, Poland; ‡ Biotechnology Center, Silesian University of Technology, Krzywoustego 8, 44-100 Gliwice, Poland; § Drug Research Progam, Faculty of Pharmacy, University of Helsinki, Viikinkaari 5E, 00014 Helsinki, Finland; ∥ Department of Genetics, ELTE Eötvös Loránd University, Pázmány P. stny. 1/C, Budapest H-1117, Hungary

## Abstract

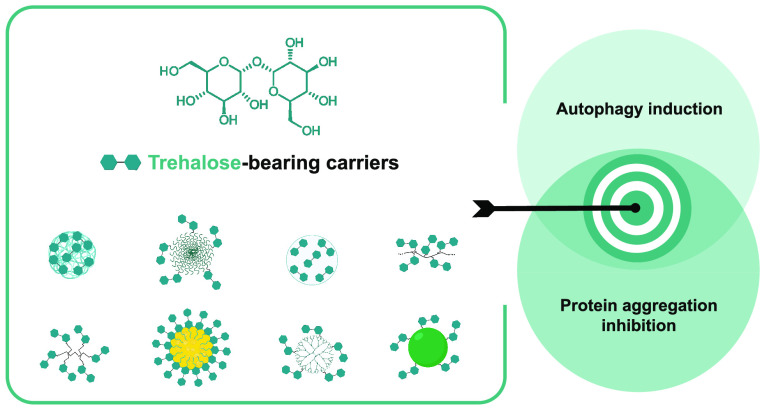

In recent years, trehalose, a natural disaccharide, has attracted growing attention because of the discovery of its potential to induce autophagy. Trehalose has also been demonstrated to preserve the protein’s structural integrity and to limit the aggregation of pathologically misfolded proteins. Both of these properties have made trehalose a promising therapeutic candidate to target autophagy-related disorders and protein aggregation diseases. Unfortunately, trehalose has poor bioavailability due to its hydrophilic nature and susceptibility to enzymatic degradation. Recently, trehalose-bearing carriers, in which trehalose is incorporated either by chemical conjugation or physical entrapment, have emerged as an alternative option to free trehalose to improve its efficacy, particularly for the treatment of neurodegenerative diseases, atherosclerosis, nonalcoholic fatty liver disease (NAFLD), and cancers. In the current Perspective, we discuss all existing literature in this emerging field and try to identify key challenges for researchers intending to develop trehalose-bearing carriers to stimulate autophagy or inhibit protein aggregation.

## 1 Significance

- Trehalose is considered a promising therapeutic candidate to combat autophagy-related disorders and diseases associated with protein aggregation.
- Given the increase in clinical trials of free trehalose and the problems associated with its poor bioavailability, it is expected that innovative strategies for the delivery of trehalose will be of great importance soon.
- Potential strategies for the development of trehalose-bearing carriers as alternatives to free trehalose have recently been the focus of extensive study.

## 2 Introduction

Trehalose is a naturally occurring homodisaccharide composed of two d-glucose units linked at their anomeric positions by an α,α′-1,1′-glycosidic bond. Trehalose is widely distributed in nature and is biosynthesized by many classes of organisms, such as bacteria, yeast, fungi, plants, and invertebrates. However, its biosynthetic pathways have not been found in vertebrates, including mammals.^1,2^ In recent years, trehalose has attracted growing attention as a promising therapeutic thanks to numerous *in vitro* and *in vivo* studies indicating its ability to stimulate autophagy.^3^ To date, the therapeutic potential of trehalose attributed to its autophagy stimulation effect has been studied for diseases such as diabetes and nonalcoholic fatty liver disease (NAFLD),^4,5^ atherosclerosis,^6,7^ and ischemic-related diseases.^8,9^ However, the main focus is to demonstrate the utility of trehalose in the treatment of neurodegenerative diseases, including Parkinson’s disease (PD),^10,11^ Lewy body dementia,^12^ Alzheimer’s disease (AD),^13^ and amyotrophic lateral sclerosis (ALS).^14^ Trehalose also exerts neuroprotection through antiaggregation effects.^15,16^ Several studies have shown that trehalose can directly maintain the protein’s structural integrity and limit the aggregation of pathologically misfolded proteins.^17^

Currently, there are several ongoing clinical trials with trehalose for the treatment of neurodegenerative diseases and other disorders where trehalose is believed to be an autophagy activator or inhibitor of protein aggregation. Recent clinical trials in various developmental stages are described in Table 1.

**Table 1:** Summary of Clinical Trials on Trehalose As an Active Ingredient^a^

disease	delivery	phase	stage	year (first posted)	clinical trial identifier	sponsor
spinocerebellar ataxia type 3	oral		recruiting	05/2020	NCT04399265	National University of Malaysia
spinocerebellar ataxia type 3	intravenous infusion	II/III	active, not recruiting	08/2022	NCT05490563	Seelos Therapeutics, Inc.
amyotrophic lateral sclerosis	intravenous infusion	II/III	recruiting	03/2020	NCT04297683	Merit E. Cudkowicz, MD
amyotrophic lateral sclerosis	intravenous infusion	II/III	enrolling by invitation	09/2021	NCT05136885	Merit E. Cudkowicz, MD
amyotrophic lateral sclerosis	intravenous infusion		available (expanded access)	10/2022	NCT05597436	Seelos Therapeutics, Inc.
acute coronary syndrome	intravenous infusion	II	unknown	09/2018	NCT03700424	Mashhad University of Medical Sciences
Alzheimer’s disease	intravenous infusion	II	withdrawn (no results posted)	04/2022	NCT05332678	Seelos Therapeutics, Inc.
Parkinson’s disease	oral	IV	not yet recruiting	05/2022	NCT05355064	Neuromed IRCCS
type 2 diabetes	oral	early I	recruiting	10/2022	NCT05593549	Medical College of Wisconsin

a Source: www.clinicaltrials.gov (accessed on 1 September 2023).

Unfortunately, the therapeutic application of trehalose has some limitations. Free trehalose has poor bioavailability because it can be readily hydrolyzed into glucose molecules by trehalose-specific enzyme, trehalase, which is found primarily in the small intestine.^2^ Moreover, without external interventions (e.g., electroporation, ultrasound, and osmotic stress), free trehalose is poorly taken up by the cells because of its strong hydrophilicity that hampers its ability to cross phospholipid bilayers of the cell membrane.^18^ Therefore, many studies suggest that trehalose should be used in relatively high doses of 100 mM (*in vitro*) and 2–3 g/kg/day and 2–4% (w/v) (*in vivo*; intraperitoneal and oral administration, respectively) to retain its potential.^3^ Oral administration of high doses of trehalose is also undesirable given the recent report linking dietary trehalose to increased prevalence of epidemic *Clostridium difficile* strains.^19^ Because of such limitations, it would be beneficial to improve the delivery of trehalose to specific target cells and tissues while also protecting trehalose from enzyme-induced hydrolysis. One of the strategies is the use of nanosized trehalose carriers in which trehalose can be incorporated either by chemical conjugation or by physical entrapment before administration. These nanocarriers have some advantages over simple trehalose conjugates, which are another possible strategy to deliver trehalose. The use of trehalose-containing nanocarriers provides protection of trehalose against enzymatic degradation, improves the systemic circulation and bioavailability of trehalose, and could improve targeting of desired cells through the attachment of targeting moieties.

**Figure 1:**
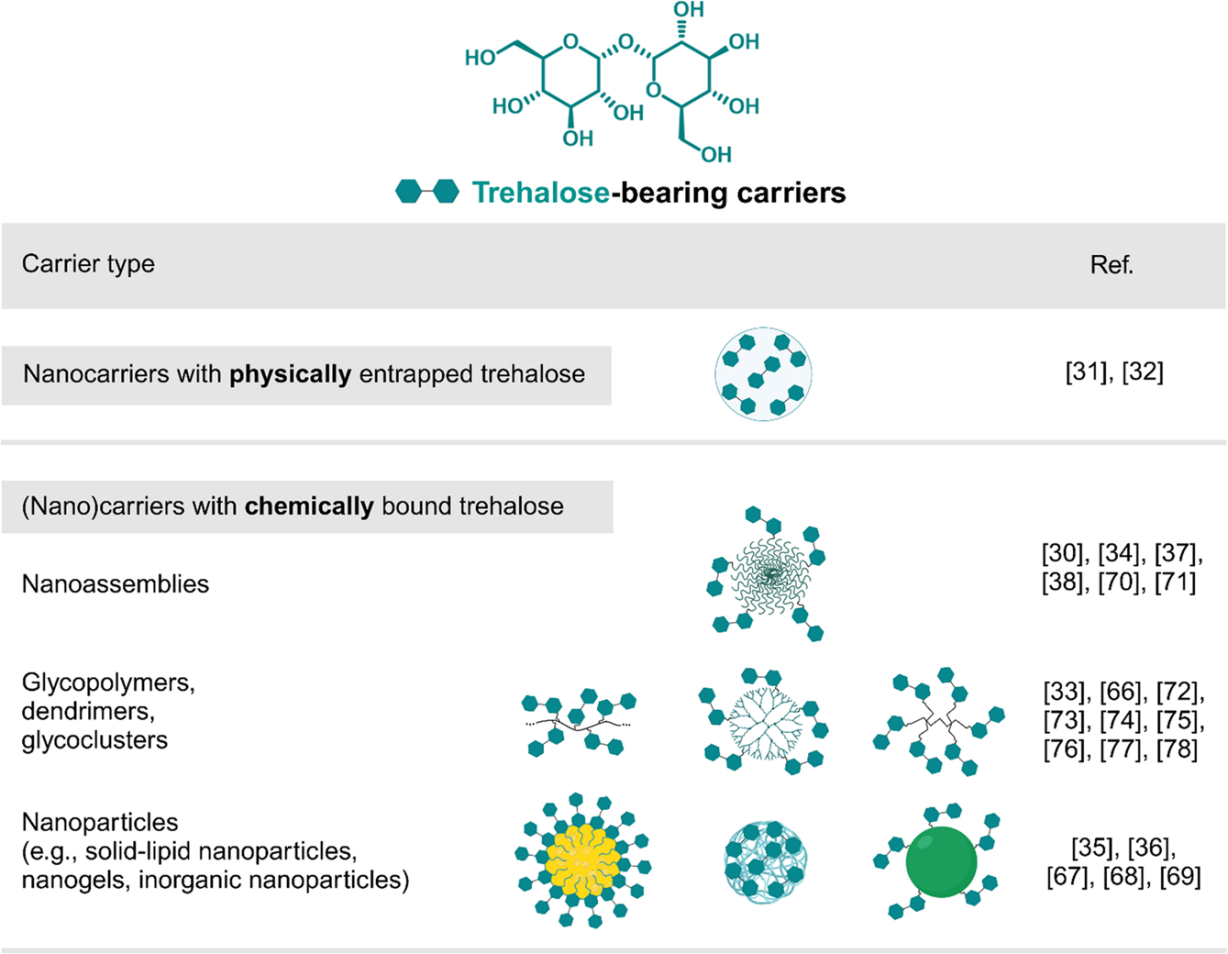
Chemical structure of trehalose and types of trehalose-bearing carriers for autophagy induction and inhibition of protein aggregation.

Trehalose-bearing carriers have been extensively developed for various biomedical applications, e.g., protein and peptide stabilization and delivery, gene delivery, bacteria-targeted applications, cell culture, and cryopreservation.^20,21^ However, the field of their utilization to target autophagy induction or inhibition of protein aggregation is still in its infancy, and just over 20 trehalose-bearing carriers have currently been developed for these purposes. Trehalose-bearing carriers targeting these two effects can be categorized into several groups: nanocarriers with physically entrapped trehalose, nanoassemblies, glycopolymers, dendrimers, glycoclusters, and nanoparticles (e.g., solid lipid nanoparticles, inorganic nanoparticles, and nanogels) (Figure 1). Moreover, the two approaches can be distinguished by the way in which trehalose is bound. In the first approach, trehalose is physically entrapped within a carrier or covalently bound through a labile bond. Thanks to this, it can be released. In the second strategy, trehalose is permanently bound to the carrier and nonreleasable, and the desired effect is studied for “poly(trehalose)” species containing multiple copies of trehalose within one macromolecule or nanoparticle.

The current Review aims to discuss all of the trehalose-bearing carriers that have been developed to stimulate autophagy or inhibit protein aggregation, as well as to clarify perspectives for this field. The number of currently ongoing clinical trials on the use of free trehalose in therapies is constantly growing, and it is likely that future research in this area will include the study of trehalose carriers as an alternative to free trehalose.

## 3 Trehalose-Bearing Carriers for Induction of Autophagy

**Figure 2:**
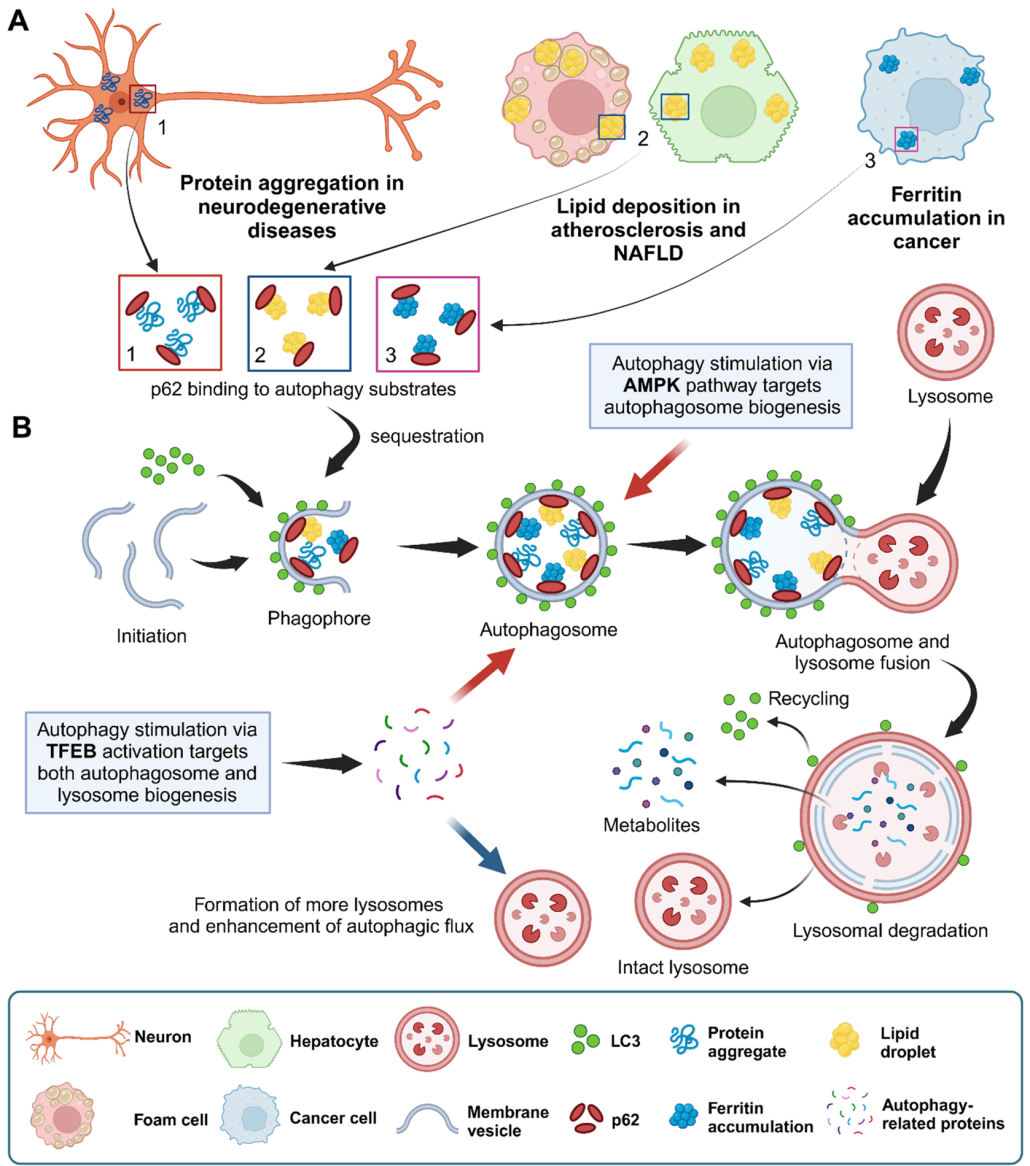
(A) Various autophagy substrates associated with impaired autophagy diseases. (B) Autophagy stimulation via the TFEB and AMPK pathways.

Autophagy is a highly conserved cellular recycling process that controls the degradation of damaged organelles and cytosolic proteins, including misfolded proteins. Many human disorders are strongly correlated with the malfunctioning of autophagy, including neurodegenerative diseases, atherosclerosis, NAFLD, and cancer.^22−26^ Targeting autophagy stimulation can be a therapeutic approach for the degradation of aggregated proteins or Lewy bodies associated with neurodegeneration, removal of oxidized lipids associated with atherosclerosis, removal of triglycerides associated with NAFLD, and clearance of accumulated ferritin associated with cancer (Figure 2A).^23,24^ Autophagy mechanisms can be categorized into three main types on the basis of how intracellular materials are transported to lysosomes, namely, microautophagy, macroautophagy, and chaperone-mediated autophagy. Macroautophagy is believed to be the main pathway with a significant catabolic potential for degrading cytoplasmic proteins and organelles. The term “autophagy” is usually used as a synonym for macroautophagy, and hereafter, when referring to the macroautophagy, we simply use the term “autophagy.”

The activation of autophagy leads to the binding of sequestosome 1 (SQSTM1/p62) to autophagy substrates, sequestration into double-membrane vesicles to form a phagophore involving microtubule-associated protein 1*A*/1B-light chain 3 (LC3), and the formation of the autophagosome (Figure 2B). The second step of autophagy is the fusion of the autophagosome with a lysosome to form the autolysosome, where the substrates can be degraded into metabolites by lysosomal proteases. Both p62 and LC3 are commonly used to quantify autophagic flux.

To date, two main pathways have been proposed by which trehalose kickstarts autophagy. The first pathway is that trehalose blocks the transport of glucose and fructose through glucose transporter family proteins (GLUTs), thereby generating a starvation like (low adenosine triphosphate) state, which in turn triggers autophagy via the activation of AMP-activated protein kinase (AMPK) and unc-51-like kinase 1 (ULK1).^5,27,28^ The second pathway is that trehalose stimulates autophagy via transcription factor EB (TFEB) activation, which results in increased expression of lysosomal hydrolases and membrane proteins and various autophagy-related components.^29^

**Figure 3:**
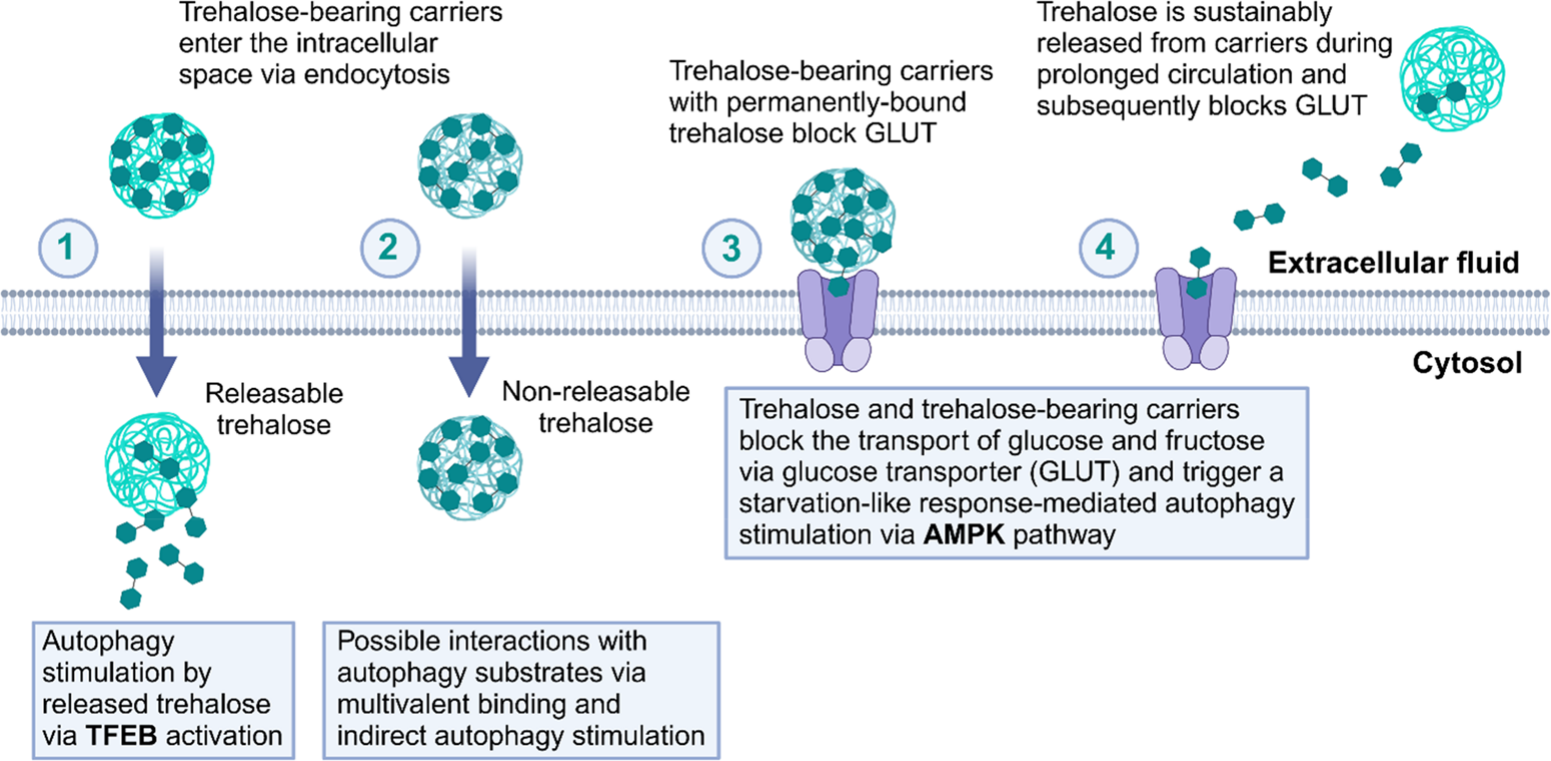
Four possible pathways through which trehalose-bearing carriers can stimulate autophagy.

It follows from the above that the mechanisms of autophagy induction involving free trehalose have been extensively studied, but there are no reports on how carriers containing trehalose could function, especially those that contain covalently bound, nonreleasable trehalose. Several mechanisms of action of trehalose-bearing carriers can be hypothesized, including (1) the carrier penetrates the cell via endocytosis and releases trehalose inside the cell, which induces autophagy via TFEB; (2) the carrier decorated with pendant and permanently bound trehalose penetrates the cell via endocytosis and interacts directly with autophagy substrates, which causes indirect stimulation of autophagy; (3) the carrier causes a multivalent interaction with GLUT and consequently blocks the transporter and stimulates autophagy via the AMPK pathway; and (4) trehalose is released from the carrier in the extracellular space and interacts with GLUT, which results in a similar effect (Figure 3). The current studies on trehalose-bearing carriers for autophagy stimulation are summarized in Table 2.

**Table 2:** Summary of Studies on Trehalose-Bearing Carriers for Autophagy Induction

carrier type	targeted disease	autophagy induction *in vitro* studies	autophagy induction *in vivo* studies	ref
trehalose, l-arginine, and phosphatidylserine-based nanomotors	atherosclerosis	expression of LC3-I, LC3-II, and p62 proteins in RAW 264.7 macrophages	antiatherosclerotic effects in ApoE^–/–^ mice	(30)
hydroxypropyl-β-CD-based oridonin and trehalose-loaded nanovesicles^a^	atherosclerosis	expression of mCherry-GFP-LC3B protein in RAW264.7 macrophages		(31)
trehalose-loaded manganese oxide-integrated mesoporous silica nanoparticles^a^	cancer	expression of LC3-I, LC3-II, and p62 proteins in PANC1 and 4T1 cancer cells	tumor growth inhibition in PANC1 tumor-bearing mice	(32)
trehalose glycopolymers with 20 or 40 pendant trehalose moieties	NAFLD	expression of AMPK(T172), LC3-I, and LC3-II proteins in mouse primary murine hepatocytes		(33)
trehalose-nucleolipid-based PLGA and solid lipid nanoparticles	neurodegeneration	expression of LC3-I and LC3-II proteins in BE(2)-M17 neuroblasts		(34)
trehalose-releasing nanogels	not specified		expression of GFP-LC3 and p62 proteins in transgenic zebrafish larvae; expression of mCherry-Atg8a and GFP-p62 proteins in transgenic *Drosophila* larvae	(35)
trehalose-coated gold nanoparticles	not specified	expression of LC3-I and LC3-II proteins in HeLa cervical cancer cells		(36)
trehalose-functionalized solid lipid nanoparticles	not specified	expression of LC3-I and LC3-II proteins in HeLa cervical cancer cells		(37)
trehalose-squalene-based nanoassemblies	not specified	expression of LC3-I, LC3-II, and mCherry-GFP-LC3B proteins in HeLa cervical cancer cells		(38)

a Trehalose is incorporated physically.

The first attempt to use trehalose-bearing carriers to stimulate autophagy came from the Seneci group. These carriers were fabricated through the conjugation of trehalose with squalene or betulinic acid and subsequent assembly into the corresponding nanoassemblies.^37^ Such nanolipid conjugates were expected to facilitate cellular internalization and then induce autophagy upon trehalose release following the disassembly and hydrolysis of the ester bond through which trehalose is bound to squalene or betulinic acid residues. Unfortunately, none of the nanoassemblies induced autophagy *in vitro*, likely because of insufficient concentration of free trehalose resulting from the limited hydrolysis of ester linkage in the cell environment. In the next attempt, squalene–trehalose conjugates were bound via a biologically labile disulfide bond.^38^ Two nanoassemblies from trehalose–monosqualene and trehalose–disqualene conjugates were fabricated. Nanoassemblies containing trehalose–disqualene conjugates demonstrated a higher efficacy in terms of autophagy induction than monosqualene analogues, free trehalose, and nonassembled precursors, as studied in LC3-overexpressed HeLa cells. According to the authors’ hypothesis, this effect can be attributed to the greater permeability through the cellular membrane of the disqualenylated nanoassemblies containing more hydrophobic cores compared with the monosqualenylated nanoassemblies. The third approach attempting autophagy induction from the Seneci group involves trehalose-decorated gold nanoparticles prepared through the reduction of gold salt in the presence of thiol-terminated polyethylene glycol (PEG)–trehalose conjugate.^36^ The trehalose–PEG gold nanoparticles cause measurable autophagy induction *in vitro* without any significant cytotoxicity in HeLa cells.^36^

**Figure 4:**
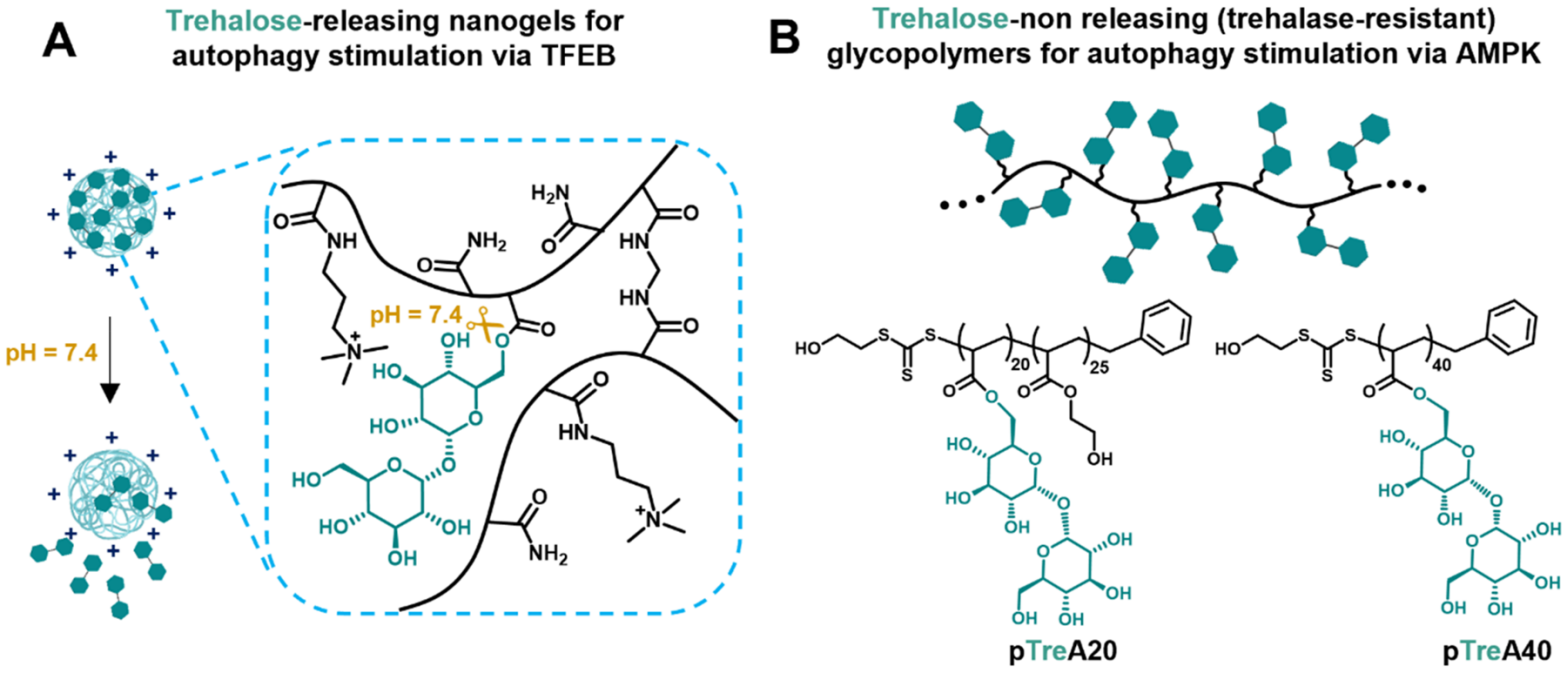
(A) Trehalose-releasing nanogels and (B) trehalose-nonreleasing glycopolymers to target two different pathways of autophagy stimulation.

Another strategy to target autophagy induction has been developed in our lab and concerns trehalose-rich nanogels with covalently attached but releasable trehalose.^35^ The nanogels are designed on the basis of the previous studies on bulk hydrogels, which have shown that specific composition of polymeric network could ensure prolonged release of trehalose under physiologically relevant conditions.^39^ As demonstrated, copolymerizing 6-*O*-acryloyl-trehalose with acrylamide-type monomers allows the fabrication of materials that can sustainably release trehalose at pH 7.4 due to the interaction of the amide protons with the ester bond in adjacent acrylate units, which strongly accelerates ester hydrolysis (Figure 4A). Employing this acrylate/acrylamide approach, a series of nanosized trehalose-releasing hydrogels is synthesized by the reverse microemulsion method through photoinitiated free radical copolymerization of 6-*O*-acryloyl-trehalose and acrylamide and/or cationic (3-acrylamidopropyl)-trimethylammonium chloride.^35^ The cationic monomer is selected to provide colloidal stability and to functionalize nanogels with positive charge, which would ensure electrostatic interactions with negatively charged membranes to enhance brain targeting via adsorptive-mediated transcytosis and make them potentially applicable to target neurodegenerative disorders. *In vivo* studies on transgenic zebrafish and *Drosophila* larvae show that cationic trehalose-releasing nanogels can significantly induce autophagy, as indicated by the increased levels of LC3 and autophagy-related protein Atg8 and downregulation of p62 levels, thereby proving their promising potential as trehalose delivery vehicles for autophagy stimulation.^35^

Recent nanosystems containing trehalose-conjugates have been fabricated by rapid mixing of amphiphilic trehalose–nucleolipid conjugates into solid lipid nanoparticles or by encapsulation of trehalose–nucleolipid conjugates into poly(lactic-*co*-glycolic acid) nanoparticles through nanoprecipitation.^34^ In both cases, thymidine-based lipids are coupled to trehalose through an ester moiety, which allows its enzymatic release in neuronal cells. *In vitro* assays in neuronal cells show efficient cellular uptake of these nanosystems and enhanced autophagy compared with those of molecular trehalose, as demonstrated by immunoblotting and transfection assays.

Autophagy plays also a crucial role in liver homeostasis, and can break down and remove hepatocellular lipid accumulation.^40,41^ Hepatic autophagy is believed to play a protective role during NAFLD and nonalcoholic steatohepatitis (NASH), which is pathologically more advanced than NAFLD. DeBosch’s group has recently investigated the effects of trehalose glycopolymers on hepatocyte CD53 blocking in basal and overnutrition contexts, which may be an effective way to reduce diseases that combine overnutrition and inflammation, such as NASH and type 2 diabetes.^33^ It is believed that free trehalose blocks carbohydrate uptake into hepatocytes as a nonselective inhibitor of GLUT.^27^ Therefore, the question arises whether polymers containing pendant trehalose can also block GLUT. One can hypothesize that trehalose glycopolymers can effectively block GLUT because of the possible multivalent interactions, that is, the so-called “carbohydrate cluster effect.”^42^ To elucidate this effect, two glycopolymers containing 20 or 40 repeating units of 6-*O*-acryloyl-trehalose were synthesized (pTreA20 and pTreA40, Figure 4B). They differed in the content and density of pendant trehalose, but their polymer backbones were identical. Both glycopolymers were tested as trehalase-resistant analogues in the *in vitro* model of NAFLD. While pTreA40 treatment in free fatty acid (FFA)-induced lipotoxicity of hepatocytes can significantly induce autophagy, as confirmed by the increased pAMPK (phosphorylated AMP-activated protein kinase) and LC3-II levels, pTreA20 can only slightly increase the autophagic activity, which is similar to free trehalose.

There are two reports in which trehalose-bearing carriers have been studied to target atherosclerosis. Atherosclerosis is a chronic disease characterized by inflammation in artery walls caused by lipid deposition that results in the formation of plaques and narrowing of blood arteries.^43^ As a result of the increased production of reactive oxygen species (ROS)-mediated oxidative stress, the cellular recycling process (autophagy) is impaired as atherosclerosis progresses.^43,44^ In the early to middle stages of atherosclerosis development, i.e., the formation of fatty streaks and intermediate lesions, autophagy is crucial for cholesterol efflux by macrophages and, thus, the reduction of foam cell formation.^45−47^ Autophagy, however, demonstrates a protective effect (promotion of plaque stability) in advanced atherosclerotic lesions by enhancing macrophage survival and inhibiting necrotic core formation.^48,49^

**Figure 5:**
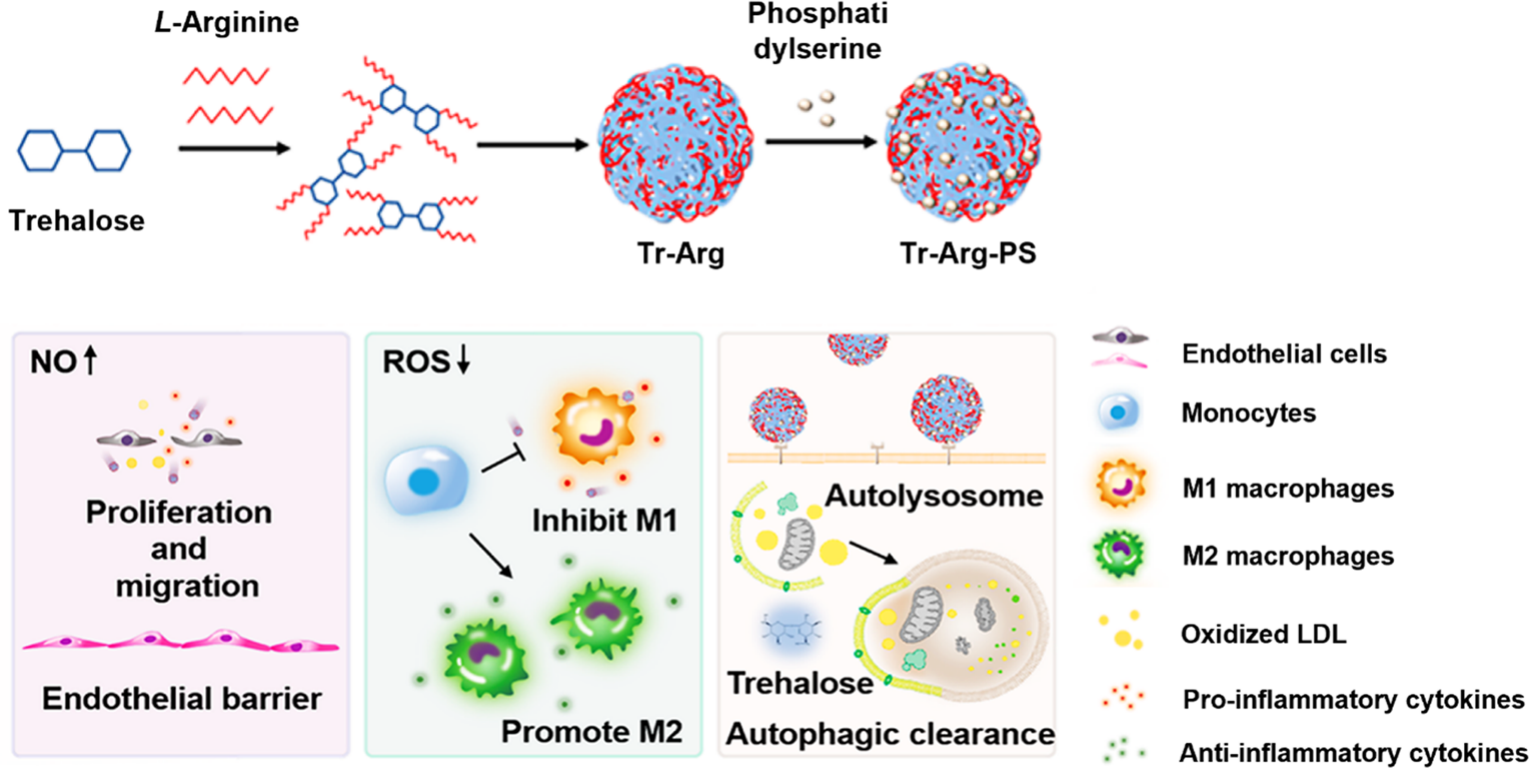
Synthesis and the antiatherosclerosis effects of Tr-Arg-PS nanomotors, including promoting endothelial repair, regulating phenotypic polarization of macrophages, and inducing macrophage autophagy. Adapted with permission from ref (30). Copyright 2022 American Chemical Society.

The first approach to use trehalose carriers to enhance autophagy in atherosclerosis is trehalose-based nanomotors.^30^ The nanomotors are constructed via double self-assembly of (i) trehalose conjugates containing four arginine molecules (Tr-Arg), and (ii) nanoparticles functionalized by phosphatidylserine, which allow the construction of Tr-Arg-PS nanomotors (Figure 5). According to the authors, the targeting mechanism of Tr-Arg-PS nanomotors is twofold. The first is to accelerate the penetration of nanomotors into the target macrophages using nitric oxide (NO) as the driving force generated by the reaction between arginine and ROS in the interstitial fluid of atherosclerotic lesions. The second is to stimulate an “eat me” signal to macrophages to enhance cell uptake. Tr-Arg-PS nanomotors can stimulate autophagy in foam cells, as confirmed by a much lower p62 protein level compared with the negative control (foam cells without any treatments), and significantly reduce lipid deposition *in vitro*. Tr-Arg-PS nanomotors can enhance the *in vivo* targeting efficiency to atherosclerotic plaques by almost double compared with nanomotors, where the l-arginine component is substituted by l-lysine and, thus, NO cannot be generated. Tr-Arg-PS nanomotors can reduce aortic lesion area by ∼20% in an atherosclerotic mice model after two months of treatments. The authors suggest that the induction of autophagy in foam cells may be due to the presence of trehalose in the nanomotors. Unfortunately, no suggestion is made as to how free trehalose can be released inside the cells from the nanomotors. Trehalose conjugates have been synthesized by substituting the sulfonated trehalose for the amino group of arginines. Therefore, it is questionable whether cleavage of the bound amino group to release free trehalose is possible inside cells.

Stimulation of autophagy to treat atherosclerosis is also targeted in the study on multiloaded self-assembled nanovesicle systems composed of amphiphilic H9 peptide and hexadecyl phosphorylcholine loaded with physically entrapped trehalose and the (HP-β-CD)/oridonin inclusion complex.^31^ The synergistic effects of oridonin and trehalose can inhibit foam cell formation in RAW264.7 cells, and can reduce inflammatory cytokines IL-1β, IL-6, and TNF-α and promote the formation of autophagosomes, as confirmed by the increased level of LC3 in foam cells.^31^

The modulation of autophagy may also play a role in cancer treatment. The role of autophagy in cancer appears to be more complicated and is influenced by tumor type, disease stage, and host factors because of its dual role as a tumor suppressor and tumor promotor.^50−52^ The concept that autophagy prevents the development of tumors is now well understood. However, once a tumor has become well-developed, autophagy processes are necessary to support uncontrolled cell proliferation and enhanced metabolic activity, thereby creating a dependence on autophagy for tumor survival.^51−53^ In early tumor development, autophagy promotes ferroptosis-mediated tumor suppression by degrading accumulated ferritin in cancer cells.^24,53^

Chen’s group has developed trehalose-loaded mSiO_2_@MnOx-mPEG nanoparticles for autophagy-enhanced cancer-cell ferroptosis.^32^ The dual mechanism of action of nanoparticles starts by inhibiting GPX4 (glutathione peroxidase 4)-induced ferroptosis in cancer cells due to the high glutathione (GSH) consumption efficiency of nanoparticles. Second, the high consumption of GSH and sensitivity to pH-mediated nanoparticle degradation lead to the release of trehalose from nanocarrier systems, which induces autophagy and further facilitates ferroptosis through the nuclear receptor coactivator 4 (NCOA4)-mediated degradation of ferritin. Bare mSiO_2_@MnOx-mPEG nanoparticles exhibit a desirable mesoporous nanostructure, which can efficiently entrap trehalose molecules. Encapsulated trehalose can be quickly released upon exposure of nanoparticles to acidic pH and a high GSH level representing tumor microenvironments. Treatment with trehalose-loaded nanoparticles in pancreatic cancer cells enhances autophagosome and autolysosome formation, as well as shrunken mitochondria and normal nuclei without chromatin condensation, which indicate autophagy and enhanced cancer ferroptosis. In addition, GPX4 and p62 protein expression can be suppressed by treatments with trehalose nanoparticles compared with the control, while LC3-II and NCOA4 protein levels are enhanced with dose-dependent effects on cancer ferroptosis. An *in vivo* study in tumor-bearing mice indicates that treatments with nanoparticles can inhibit tumor growth by ∼90% over 2 weeks of treatments compared with both free trehalose and negative control. These findings imply that nanoparticles boost the efficacy of trehalose for cancer treatment.

## 4 Trehalose-Bearing Carriers for Inhibition of Protein Aggregation

Polypeptides/proteins fold through different intermediates into their functional, native, three-dimensional conformation. At times, certain factors, such as environmental stress, mutations, or translational errors, can cause protein misfolding. Misfolded proteins can be refolded to their native states or degraded by different cellular mechanisms. However, if these mechanisms fail, misfolded proteins can aggregate to form amorphous aggregates or assemble through prefibrillar species into highly ordered, β-sheet-rich aggregates with fibrous morphology called amyloids.^54^ Such pathogenic amyloids can then form extracellular plaques or intracellular inclusions, which affects the healthy function of tissues and organs, and their increased accumulation is associated with the development of numerous incurable human disorders, both neurodegenerative and non-neuropathic.^55−57^ Some examples of human polypeptides/proteins that possess an inherent tendency to form amyloid fibrils and corresponding disorders include α-synuclein in PD, dementia with Lewy bodies (DLB), multiple system atrophy (MSA), amyloid-β (Aβ) and tau in AD, mutant huntingtin in Huntington’s disease (HD), ubiquitin in ALS, islet amyloid polypeptide (IAPP) in type 2 diabetes, or lysozyme in systemic lysozyme amyloidosis. Apart from endogenous proteins, several pharmaceutical polypeptides and proteins are also known to have a high propensity to form amyloid-like fibrils. An example is recombinant insulin, in which long-term subcutaneous administration can result in the development of localized insulin-derived amyloidosis at the injection sites. Stimulation of autophagy to disintegrate the formed amyloid deposits is one of the strategies that are developed to combat disorders associated with protein aggregation.^26,58^ Another strategy targets amyloid formation and includes prevention of protein aggregation at an early stage.^59^ Among many types of studied compounds, numerous low-molecular-weight natural saccharides, including trehalose, are identified as chemical chaperones, which can suppress protein aggregation.^60−65^ Unfortunately, considering the high concentration required to observe the desired effect, the antiamyloidogenic efficiency of saccharides is rather low. Some recent studies have found that this efficiency can be significantly amplified by creating synthetic structures bearing multiple copies of saccharides. For example, glycoclusters prepared by installing six trehalose, lactose, galactose, or glucose residues on a dipentaerythritol core significantly overperform compared with the corresponding mono- or disaccharides in retarding the formation of Aβ40 fibrils.^66^ Particularly, the trehalose glycocluster causes an extremely significant retardation. Moreover, the trehalose glycocluster can protect neurons from Aβ40-induced cell death, although this neuroprotective activity is similar to free trehalose. The enhanced performance of trehalose-bearing carriers over the molecular trehalose in interfering with protein aggregation has also been demonstrated for several trehalose-bearing glycopolymers, dendrimers, nanoparticles, and nanoassemblies, and they are overviewed in Table 3.

**Table 3:** Summary of Studies on Trehalose-Bearing Carriers for Inhibition of Protein Aggregation

targeted disease	carrier type	model protein^a^	protein aggregation *in vitro* or *in vivo* studies	ref
neurodegenerative diseases	trehalose glycocluster with six pendant trehalose moieties	Aβ(1–40)	cytotoxicity of Aβ(1–40) aggregates toward mouse primary cortical neurons	(66)
	iron oxide nanoparticles coated with trehalose-containing polymeric network	lysozyme, Aβ(1–40)	aggregation of mutant huntingtin inside HD150Q neuronal cells; aggregation of mutant huntingtin in HD transgenic mice	(67)
	trehalose-based nanoparticles from hydrothermal carbonization	lysozyme, Aβ(1–40), insulin	aggregation of mutant huntingtin inside HD150Q neuronal cells; cytotoxicity of mutant huntingtin aggregates toward HD150Q neuronal cells; cytotoxicity of lysozyme amyloids toward CHO ovarian cells	(68)
	trehalose-coated gold nanoparticles		aggregation of mutant huntingtin inside HD150Q neuronal cells; cytotoxicity of mutant huntingtin aggregates toward HD150Q neuronal cells	(69)
	trehalose-functionalized biodegradable polylactide nanoparticles		aggregation of mutant huntingtin inside HD150Q neuronal cells; cytotoxicity of mutant huntingtin aggregates toward HD150Q neuronal cells; ROS generation inside lysozyme fibrils-treated HT22 mouse hippocampal neuronal cells	(70)
	trehalose-functionalized biodegradable polycarbonate-*co*-polylactide nanoparticles	Aβ(1–42)	cytotoxicity of Aβ(1–42) aggregates toward SH-SY5Y neuroblastoma cells; ROS generation inside Aβ(1–42) oligomers-treated SH-SY5Y neuroblastoma cells	(71)
	trehalose-terminated polyglycerol dendrimers	lysozyme	aggregation of mutant huntingtin inside HD150Q neuronal cells	(72)
	trehalose glycopolymers of 6-acrylamido-6-deoxy-trehalose and acrylamide	Aβ(1–40)	cytotoxicity of Aβ(1–40) aggregates toward HeLa cervical cancer cells	(73)
	trehalose glycopolymers of 6-*O*-vinyladipoyl-trehalose or 6-*O*-vinylsebacoyl-trehalose	Aβ(1–42)	cytotoxicity of Aβ(1–42) aggregates toward HeLa cervical cancer cells	(74)
localized insulin-derived amyloidosis	trehalose glycopolymers of 6-*O*-acryloyl-trehalose	insulin		(75)
	trehalose glycopolymers of various trehalose vinylbenzyl ether regioisomers	insulin		(76)
	trehalose glycopolymers of 6-*O*-methacryloyl-trehalose	insulin		(77)
	trehalose glycopolymers with poly(lactide), poly(carbonate), or poly(caprolactone) backbone	insulin		(78)

a Model protein used in aggregation studies under fibrillation-accelerating conditions in solution.

**Figure 6:**
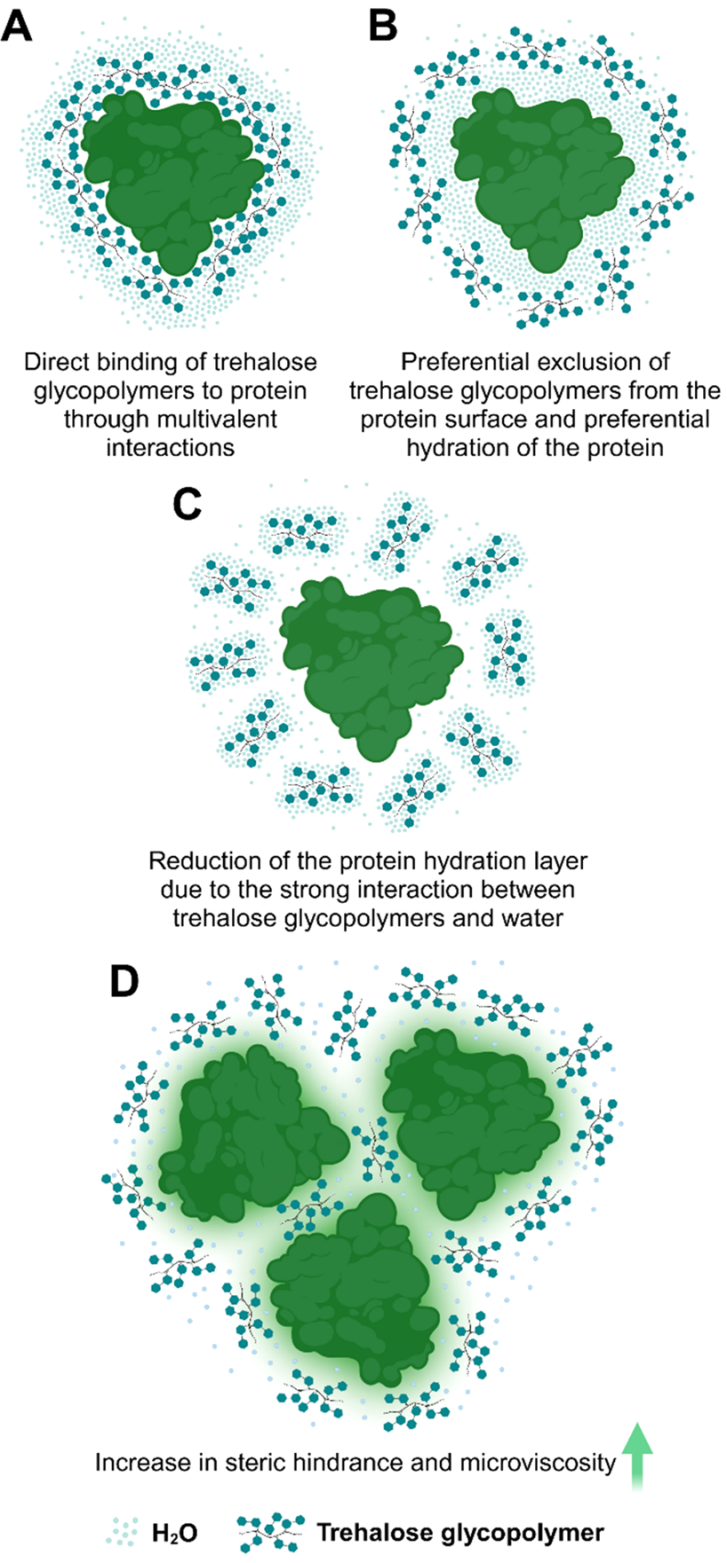
Possible mechanisms of the inhibition of protein aggregation by trehalose-bearing carriers (represented by trehalose glycopolymers).

The molecular mechanism explaining how trehalose-bearing carriers prevent proteins from aggregation into amyloid fibrils has not been studied, but on the basis of the various mechanisms postulated for trehalose, as well as for macromolecules/nanostructures,^79−81^ at least four mechanisms can be hypothesized (Figure 6). The presence of multiple copies of trehalose within one species offers the possibility of binding via multiple binding points compared with the monovalent binding of a single trehalose molecule. Thus, the first mechanism comprises direct binding of “poly(trehalose)” to the protein enhanced by multivalent interactions (Figure 6A). In this way “poly(trehalose)” can stabilize a protein’s structure but it can also prevent proteins from interacting with each other’s. Alternatively, the “poly(trehalose)” can act indirectly by being preferentially excluded from the immediate vicinity of proteins, thus promoting the preferential hydration of the protein molecules, which increases their stability (Figure 6B). It is also possible that the antiaggregation effect is the result of the kosmotropic nature of trehalose, wherein stronger interactions between “poly(trehalose)” and water molecules than between the water molecules themselves reorganize the regular water structure, which causes a reduction in the hydration layer of proteins, thus enhancing intramolecular interactions (Figure 6C). Finally, antiamyloidogenic action of trehalose-bearing carriers can be attributed to their nano/macromolecular structure and results from an increase in steric hindrance in the solution and microviscosity, which restrict protein–protein interactions and limit protein movements (Figure 6D).

**Figure 7:**
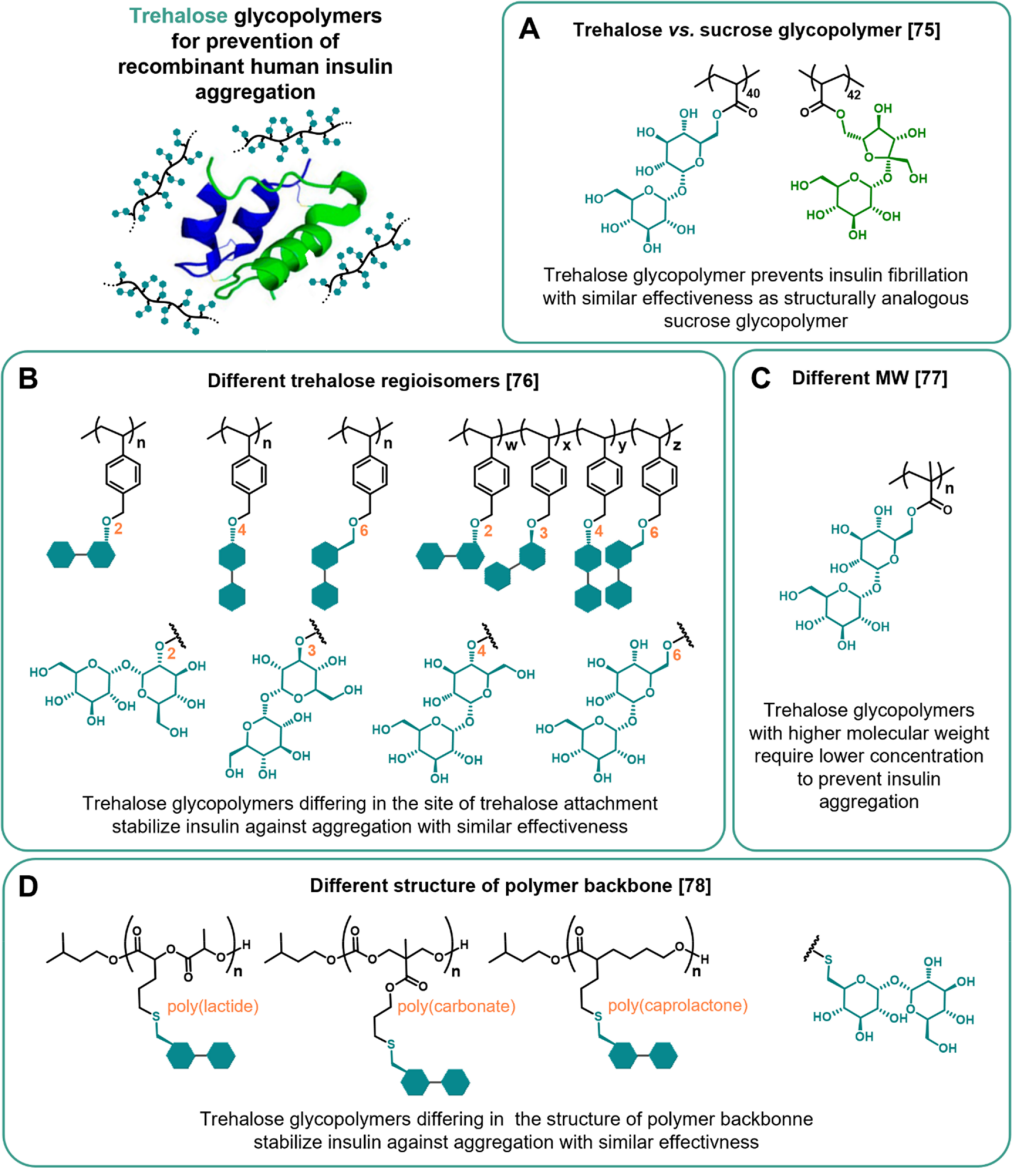
Overview of the studies on trehalose glycopolymers for the prevention of recombinant human insulin aggregation. Comparison between: (A) structurally analogous poly(6-*O*-trehalose acrylate) and poly(6-*O*-sucrose acrylate); (B) glycopolymers of different trehalose vinylbenzyl ether regioisomers; (C) poly(6-*O*-trehalose methacrylate)s with different molecular weight (MW); and (D) glycopolymers with poly(lactide), poly(carbonate), or poly(caprolactone) backbone.

The results from the study of Miura and co-workers on Aβ(1–42) and Aβ(1–40) fibrillation have shown that trehalose glycopolymers prepared from 6-*O*-vinyladipoyl-trehalose^74^ or 6-acrylamido-6-deoxy-trehalose^73^ exhibit superior antiamyloidogenic properties over glycopolymers of maltose or lactose and their corresponding disaccharide alcohols maltitol or lactitol. Furthermore, Aβ aggregates formed in the presence of trehalose glycopolymers have no cytotoxicity,^73^ or their cytotoxicity is reduced compared with aggregates formed without any additives.^74^ However, the effects are strongly affected by the structural features of the glycopolymers, e.g., the linker length between polymeric chains and sugar moieties. For example, the polymer with a shorter adipoyl linker [poly(6-*O*-vinyladipoyl-trehalose)] shows a strong aggregation inhibition effect compared with that of molecular trehalose, while poly(6-*O*-vinylsebacoyl-trehalose) with a longer alkyl side chain induces amyloid formation. As concluded, not only the structure of pendant saccharide but also the glycopolymer’s amphiphilicity play important roles in amyloid formation and inhibition. The counterparts for trehalose glycopolymers employed in these studies exhibit some structural differences, thus rendering them not perfectly comparable. They are also synthesized via noncontrolled polymerization techniques, and therefore, the uniformity in terms of molecular weight and dispersity among the compared glycomacromolecules cannot be guaranteed. Considering that the structural features of polymers can influence their effectiveness, the proper comparative study requires high structural similarity between counterparts regarding their molecular weight and dispersity, as well as the linking motif between the polymer chain and saccharide moiety. Following this direction, very recently, our group has presented a strategy for obtaining highly structurally comparable glycopolymers of trehalose and sucrose through reversible addition–fragmentation chain transfer (RAFT) polymerization of trehalose and sucrose acrylate analogues: 6-*O*-acryloyl-trehalose and 6-*O*-acryloyl-sucrose (Figure 7A).^75^ The glycomonomers share several structural commonalities, including the same molecular weight, number of hydroxyl groups, functionalization with an acryloyl moiety on the primary hydroxyl group, and nonreducing character, as well as the enablement to afford polymers with terminal α-d-glucopyranosyl moieties. As evaluated on recombinant human insulin, the studied glycopolymers demonstrate significantly amplified antiamyloidogenic performance over their corresponding molecular saccharides. The effects are concentration-dependent and are particularly prominent for higher concentrations at which glycopolymers not only retard fibrillation but also significantly decrease the amount of aggregated insulin and result in the formation of significantly shorter fibrils. Interestingly, both trehalose and sucrose glycopolymers give similar results indicating that antiamyloidogenic effectiveness is not particularly superior for trehalose decoration of the polymer, at least in the studied case.

Trehalose-bearing macromolecules have been extensively developed in the Maynard group,^20^ and part of the research has been devoted to the study of various trehalose glycopolymers for stabilization of pharmaceutical proteins/polypeptides, including human recombinant insulin. Although the studies are not focused directly on the fibrillation and amyloid fibrils formation but rather on general stability against aggregation caused by heating and agitation, the results obtained provide several important conclusions regarding the influence of glycopolymer structure on their stabilization effect. With high probability, the results are also valid in view of fibrillation. They are also valuable to be included in the current discussion, because of the widespread use of pharmaceutical insulin and the recognized risk of the development of localized amyloidosis associated with its administration. For example, the group has studied the effect of trehalose positional modification on glycopolymer’s effectiveness by comparing polymers of various trehalose vinylbenzyl ether regioisomers (modified at 2-*O*, 3-*O*, 4-*O*, or 6-*O* position) (Figure 7B).^76^ The differences in their effectiveness might potentially arise from their different conformational flexibilities, as determined by computational calculations. Unlike molecular trehalose at an equivalent concentration, all polymers inhibit insulin aggregation, but there are no significant differences between them. Despite the difference in conformational flexibility, all of the regioisomers retained the native clamshell conformation of trehalose. This is suggested to be more important for stabilization, which explains why no differences are observed between regioisomers. Moreover, it turns out that stabilization effectiveness depends on the molecular weight, as recently found by studying poly(6-*O*-trehalose methacrylate)s.^77^ Longer polymers require a lower concentration to completely prevent insulin aggregation (Figure 7C). Further development in the group has extended the library of trehalose glycopolymers by hydrolytically degradable macrostructures by changing the synthetic approach from radical polymerization of trehalose glycomonomers to postpolymerization modification. Specifically, thiolated trehalose is installed through radical-initiated thiol–ene reaction on allyl-substituted poly(lactide), poly(carbonate), or poly(caprolactone) (Figure 7D).^78^ All of these glycopolymers stabilize insulin against aggregation with fairly similar effectiveness, thereby suggesting that the presence of trehalose is more significant than the structure of the backbone. It is hypothesized that the enhanced stabilizing properties of trehalose glycopolymers are strongly related to their nonionic surfactant character, i.e., hydrophilic side moieties with a hydrophobic backbone.

**Figure 8:**
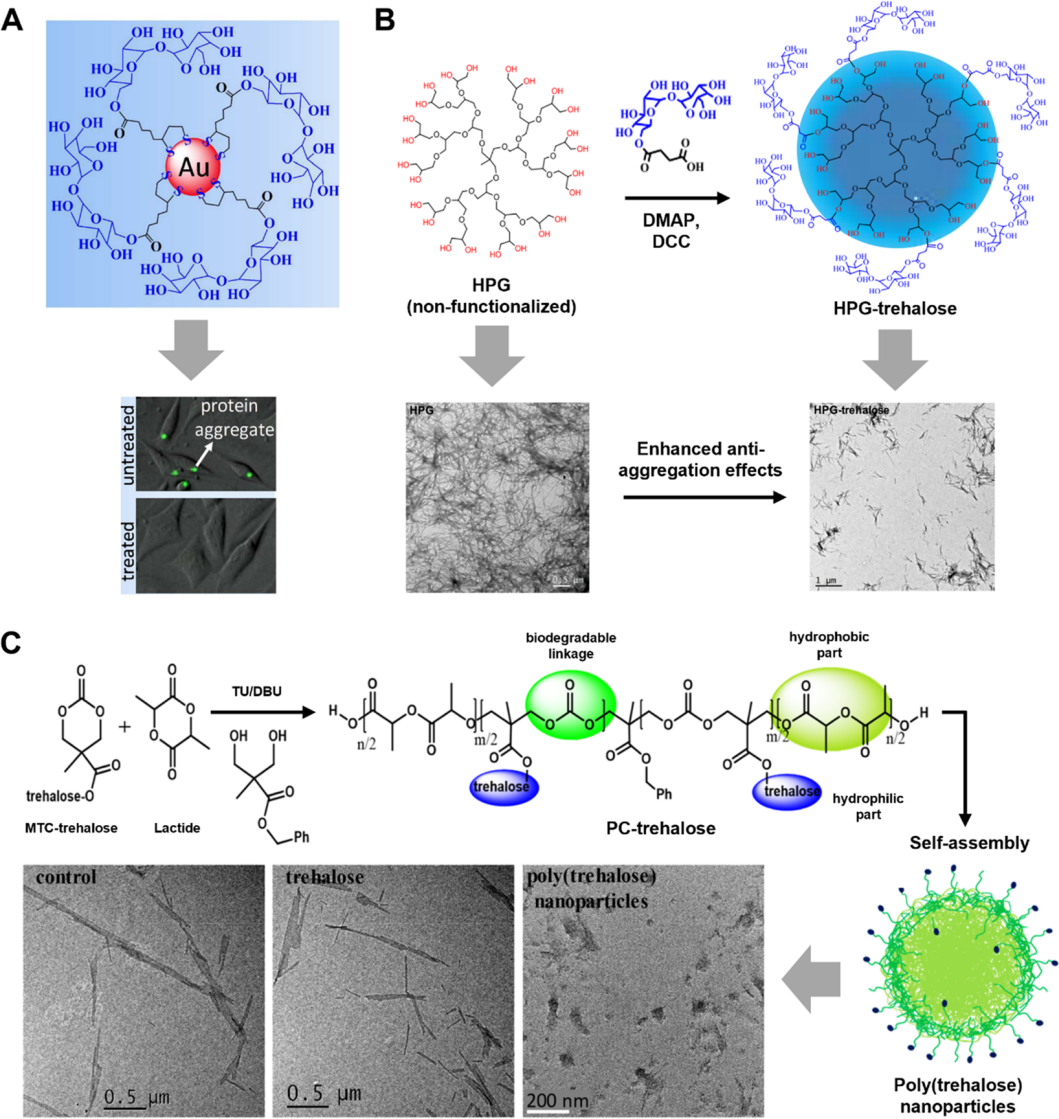
Overview of selected trehalose-bearing carriers developed in the Jana and Jana group for the inhibition of protein aggregation. (A) Trehalose-functionalized gold nanoparticle that can inhibit aggregation of polyglutamine-containing mutant proteins inside the neuronal cells. Adapted with permission from (69). Copyright 2017 American Chemical Society. (B) Synthetic pathway of trehalose-terminated hyperbranched polyglycerol dendrimers (HPG-trehalose) and its effect on preventing lysozyme fibrillation. Adapted with permission from (72). Copyright 2020 American Chemical Society. (C) Schematic pathway of trehalose-containing amphiphilic polycarbonate-*co*-polylactide copolymer (PC-trehalose) and its self-assembly into poly(trehalose) nanoparticles for inhibition of Aβ fibrillation. Adapted with permission from (71). Copyright 2023 American Chemical Society.

In recent years, antiamyloidogenic activity has also been proven by the Jana and Jana group for several poly(trehalose)-type nanoparticles.^82^ The first nanoparticles comprise plate-shaped nanoparticles decorated with trehalose, which are fabricated through the simple hydrothermal carbonization.^68^ Other nanoparticles are based on a gold^69^ or iron oxide^67^ core and decorated with trehalose by using trehalose derivatives. Specifically, gold nanoparticles are engineered through the reduction of gold salt in the presence of a trehalose–lipoic acid derivative (Figure 8A).^69^ In turn, iron oxide-cored nanoparticles are covered with polymeric coating containing trehalose fabricated through the polymerization of crotonylated trehalose, PEG-acrylate, and sulfoacrylate and/or amino-acrylate monomers. The presence of anionic, zwitterionic, or cationic units can be regulated by their ratio in order to improve cellular uptake and facilitate blood–brain barrier crossing.^67^ The last type of poly(trehalose)-type nanoparticles developed in the group include polymer-based nanoparticles formed by self-assembly of trehalose-containing polylactide^70^ or polycarbonate-*co*-lactide.^71^ These two self-assemblies differ in the composition of the polymer backbone, but more importantly, they also differ in the localization of trehalose within the polymer chain. Polycarbonate-*co*-polylactide self-assemblies are based on polymers containing pendant trehalose moieties, which were obtained from trehalose-bearing cyclic carbonate monomer (Figure 8C).^71^ In turn, in polylactide self-assemblies, trehalose is localized at the end of polymeric chains because of being used as an initiation site in ring-opening polymerization. Additionally, polylactide-based nanoparticles are enriched with dopamine-terminated or arginine-terminated polylactide. In such a design, dopamine should provide the dopamine receptor-based neuron cell uptake, arginine should enhance cellular uptake because of its cationic charge, and trehalose should provide interactions with the aggregating protein. Unlike the previous nanoparticles, both self-assemblies are biodegradable, which is highly beneficial for *in vivo* applications because it will prevent potential long-lasting accumulation in the body. The performance of the colloidal nanoparticles developed by the Jana and Jana group in both *in vitro* and *in vivo* studies on inhibiting protein aggregation can be summarized as follows. The preliminary study on accelerated in-solution fibrillation of model amyloid-forming peptides/proteins, including lysozyme,^67,68^ insulin,^68^ Aβ(1–40),^67,68^ and Aβ(1–42),^71^ proved that these poly(trehalose) nanoparticles significantly outperform free trehalose in inhibiting amyloid fibrils formation. Some of them are also able to disintegrate preformed mature amyloid fibrils into smaller parts.^67,71^*In vitro* studies on the model neuronal cell line for HD (HD150Q) have proved that the poly(trehalose) nanoparticles have high cellular uptake,^67−71^ as well as inhibit the intracellular aggregation of mutant huntingtin protein inside these cells,^67−70^ and can strongly outperform free trehalose and trehalose-absent nanoparticles (if studied). In addition, nanoparticles can reduce the amyloidogenic cytotoxicity of amyloid fibrils of mutant huntingtin aggregates toward HD150Q neuronal cells,^68−70^ lysozyme amyloids toward CHO ovarian cells^68^ and Aβ(1–42) aggregates toward SH-SY5Y neuroblastoma cells.^71^ As revealed in an intracellular ROS generation study inside Aβ(1–42) oligomers-treated SH-SY5Y neuroblastoma cells,^71^ Aβ(1–42) oligomer-treated cells produce intense intercellular ROS, while ROS generation is slightly reduced in the presence of trehalose and completely absent in cells pretreated with the poly(trehalose) nanoparticles. The authors hypothesize that the poly(trehalose) nanoparticles bind to Aβ(1–42) oligomers through multivalent interactions and reduce interactions between Aβ(1–42) oligomers and the cell membrane, thus preventing damage and cellular stress. Although the results from *in vitro* studies are encouraging, there is only one insight into the *in vivo* antiamyloidogenic effectiveness of trehalose-bearing carries, and it comes from the study on the HD-mouse model treated with zwitterionic poly(trehalose) nanoparticles containing iron oxide core.^67^ The study has revealed that, upon intravenous administration of these nanoparticles, the number of mutant huntingtin aggregates in the brain is remarkably diminished, and their action seems to be more effective than that of trehalose-absent nanoparticles.

Besides the research on trehalose-containing nanoparticles, the Jana and Jana group has also carried out a study on the antiamyloidogenic potential of trehalose-decorated hyperbranched polyglycerol dendrimers.^72^ Dendrimers are synthesized through the simple coupling of carboxylated trehalose to terminal hydroxyls (Figure 8B). Through the study of lysozyme as a model amyloidogenic protein, the trehalose-terminated dendrimers have been found to have significantly better fibril-inhibiting ability than free trehalose and to also be more effective than nonfunctionalized hyperbranched polyglycerol dendrimers. Similarly to the poly(trehalose) nanoparticles, the trehalose-terminated dendrimers are able to enter neuronal cells, thereby reaching their cytoplasm and significantly hindering intracellular aggregation of mutant huntingtin.

## 5 Conclusions and Perspectives

Trehalose-bearing carriers have emerged as superior alternatives to trehalose alone to induce autophagy or inhibit protein aggregation. The studies have successfully demonstrated the ability of these carriers to achieve improved efficacy with significantly lower amounts of trehalose. Trehalose-bearing carriers are shown to effectively interfere with the aggregation of several proteins/peptides in solution, including Aβ(1–40 and 1–42), insulin, and lysozyme, and strongly suppress intracellular aggregation of mutant huntingtin inside neuronal cells. Surprisingly, while numerous studies have demonstrated the promising antiaggregation efficacy of trehalose-bearing carriers in relevant *in vitro* or *in vivo* neurodegenerative disease models, only one study is focused on recognizing their potential against neurodegenerative diseases through autophagy induction. Other autophagy-stimulating studies have addressed the treatment of atherosclerosis, cancer, or NAFLD or only look at the potential effects of autophagy in nondisease-specified *in vitro/in vivo* models. There is also a possibility that the carriers can have synergistic proautophagy and antiaggregation actions, especially to combat neurodegenerative diseases, that remain to be verified in future studies. Most of the discussed reports were published recently (2017 or later), and the field is still in its infancy. There are many unknowns to be elucidated and some limitations and challenges to address and overcome. There is also plenty of room for developing innovative trehalose-bearing carriers.

In the majority of the designed approaches, trehalose is chemically modified to become a part of the carrier. Proper functionalization of trehalose usually requires a troublesome, multistep synthesis and limits the preparation to large quantities. Thus, before trehalose-bearing carriers can be introduced into the clinic, the synthetic methodologies need to be improved. This is not an issue for carriers in which free trehalose is physically entrapped inside. Although the entrapment of free trehalose inside carriers may seem to be easier in preparation, surprisingly, only two such types of carriers have been developed so far. The reason may be the lack of any charge on trehalose, its small size, and good water solubility, which make it challenging to keep trehalose inside the carrier and prevent its premature release before reaching the targeted area. An undeveloped strategy for fabricating carriers with releasable trehalose includes trehalose entrapment via supramolecular forces, such as specific saccharide–lectin interactions or dynamic covalent complexation into cyclic boronate esters. Trehalose release from such carriers could be triggered by competitive displacement with free sugar molecules, e.g., glucose.

Trehalose moieties on glycopolymers are accessible for specific interactions with proteins through terminal α-d-glucopyranosyl units (as has been shown on the example of interactions with concanavalin A),^75^ and it renders trehalose-bearing carriers potentially “recognizable” by cells. While on the one hand, it might be favorable to improve their cellular uptake through enhanced interactions with cell surface proteins or even directly be responsible for their action, on the other hand, they probably could interact similarly not only with targeted cells but also with healthy cells and potentially affect their functions. Thus, the *in vivo* safety and metabolic fate of trehalose-bearing carriers require a thorough examination. Generally, to fully confirm the proautophagic and antiaggregation effectiveness of trehalose-bearing carriers, more *in vivo* studies are required as most of the current results come from the research on *in vitro* models. Given that trehalose has the highest therapeutic potential for the treatment of neurodegenerative disorders, future research on trehalose-bearing carriers should also focus on effective brain targeting and blood–brain barrier crossing. The capability to integrate several functionalities within a single nanoparticle or macromolecular structure, as well as widely tailorable properties, makes targeted delivery utilizing carriers an especially promising approach.

The mechanism of action is still ambiguous for free trehalose and it is even more unknown for trehalose-bearing carriers. Usually, trehalose is permanently bound with the carrier, and the effect is studied for poly(trehalose)-type species. Given that the carriers are heavily decorated with trehalose, which makes them susceptible to multivalent interactions, it is highly likely that their mechanism of action may be completely different from that of free trehalose. The mechanism might also differ between various carriers depending on properties, such as the carrier size, its amphiphilicity, trehalose attachment position and linking motif, its incorporation density, or trehalose accessibility for interactions. Understanding the exact mechanisms and influence of the carriers’ characteristics on their action would enable the rational design of trehalose-bearing carriers. Among all of the publications on trehalose-bearing carriers, only a few of them try to explain or hypothesize possible mechanisms of their action in inducing autophagy or inhibiting protein aggregation.

Finally, careful analysis of the publications discussed suggests some thoughts concerning experimental issues. In some studies, the lack of sufficient controls impedes our ability to definitively attribute the observed effect exclusively to the presence of trehalose as opposed to a potential influence from the carrier itself. Thus, it is recommended to use not only free trehalose but also carriers that do not contain or release trehalose or counterpart carriers containing other saccharides as another control. Next, protein fibrillation in solution is extremely dependent on concentration and external conditions, such as temperature, pH, speed of agitation, exposure to air–water interfaces, etc. Thus, uniform protocols for studying protein fibrillation are necessary to compare the results obtained for various trehalose-bearing carriers in different laboratories in order to enable identification of the most promising approach and the design of future research directions. Moreover, it should always be well evidenced that trehalose has, indeed, been conjugated as depicted, and at least ^1^H and ^13^C NMR spectra with an assignment of clue signals should be provided. Finally, when claiming that conjugated trehalose can be released from the carrier under physiological conditions, this possibility should be confirmed at least under stimulated conditions.

In summary, taking into account the still growing interest in therapeutic use of trehalose to target autophagy-related disorders and diseases associated with protein aggregation, it is expected that the coming years will also bring new studies on trehalose-bearing carriers. It would be highly desirable if future research could provide better understanding of how exactly trehalose-bearing carriers induce autophagy and affect protein fibrillation, as well as provide more insights on their *in vivo* efficacy and safety. The effort should also be put toward selective delivery, particularly effective brain targeting and blood–brain barrier crossing, for the treatment of neurodegenerative disorders.

## Abbreviations

Aβ: amyloid-β
AMPK: AMP-activated protein kinase
GLUT: glucose transporter
GSH: glutathione
HD: Huntington’s disease
LC3: microtubule-associated protein 1*A*/1B-light chain 3
NAFLD: nonalcoholic fatty liver disease
ROS: reactive oxygen species
SQSTM1/p62: sequestosome 1
TFEB: transcription factor EB
Tr-Arg-PS: trehalose-based nanomotors bearing arginine and phosphatidylserine
